# Race, Sex, and Ejection Fraction-Based Differences in Transthyretin Amyloid Cardiomyopathy (ATTR-CM) Risk Prediction

**DOI:** 10.3390/jcm13206150

**Published:** 2024-10-16

**Authors:** Michel Chedid El Helou, Mohak Gupta, Muzna Hussain, Mazen Hanna, Vanessa Blumer, Preethi William, Milind Y. Desai, Bryan Q. Abadie, Lauren Ives, W. H. Wilson Tang, Wael A. Jaber, Patrick Collier, Trejeeve Martyn

**Affiliations:** 1 Department of Cardiovascular Imaging, Heart, Vascular and Thoracic Institute, Cleveland Clinic, Cleveland, OH 44195, USA; michel.chedidhelou@gmail.com (M.C.E.H.); muzna.hu@gmail.com (M.H.); desaim2@ccf.org (M.Y.D.); abadieb@ccf.org (B.Q.A.); jaberw@ccf.org (W.A.J.); colliep@ccf.org (P.C.); 2 Department of Internal Medicine, Cleveland Clinic, Cleveland, OH 44195, USA; guptam11@ccf.org; 3 Kaufman Center for Heart Failure Treatment and Recovery, Heart, Vascular and Thoracic Institute, Cleveland Clinic, Cleveland, OH 44195, USA; hannam@ccf.org (M.H.); vanessa.blumer@inova.org (V.B.); wiliap18@ccf.org (P.W.); ivesl@ccf.org (L.I.); tangw@ccf.org (W.H.W.T.)

**Keywords:** transthyretin cardiac amyloidosis, ATTR-CM score, Tc-99m pyrophosphate scintigraphy, risk prediction, population health

## Abstract

**Background:** The early detection of transthyretin cardiac amyloidosis (ATTR-CM) is essential, with Tc-99m pyrophosphate scintigraphy (PYP scan) being a key diagnostic tool. Although a previously validated score has shown promise in predicting PYP scan positivity among patients with HFpEF, further evaluation in diverse cohorts is necessary. **Objectives:** To assess the effectiveness of the ATTR-CM score in predicting PYP scan positivity within our patient population. **Methods:** We analyzed patients referred for PYP with SPECT at the Cleveland Clinic from January 2012 to January 2020, all of whom had undergone echocardiography within the previous year. The ATTR-CM score was determined using the following criteria: Age (60–69, +2; 70–79, +3; ≥80, +4), sex (male, +2), hypertension (present, −1), left ventricular ejection fraction (LVEF <60%, +1), posterior wall thickness (≥12 mm, +1), and relative wall thickness (>0.57, +2). A score of ≥6 indicated high risk. **Results:** Among the 540 patients (32% female, 33% black), 27% had an LVEF <40%. The score demonstrated good discrimination by AUC, with consistent performance across different racial groups, sexes, and LVEF categories. For scores ≥6, sensitivity was lower in women and black patients; however, lowering the cutoff to 5 markedly improved sensitivity. **Conclusions:** The ATTR-CM score displayed consistently good performance by AUC across our cohort, including patients with HFrEF. Nevertheless, its sensitivity was reduced in black patients and women. Efforts to scale ATTR-CM diagnosis tools should be mindful of demographic differences in risk prediction models.

## 1. Introduction

Transthyretin amyloid cardiomyopathy (ATTR-CM) is an increasingly recognized cause of heart failure (HF) in adults, characterized by the deposition of amyloid fibrils in the myocardium [1]. Early and accurate diagnosis of ATTR-CM, as well as distinguishing it from other causes of cardiomyopathy, is crucial for initiating appropriate treatment strategies [2]. The availability of disease-modifying treatments [3] and growing awareness of this condition have led to an emphasis on earlier suspicion and diagnosis of ATTR-CM [4]. In recent years, Technetium 99m pyrophosphate (99mTc-PYP) single-photon emission-computed tomography has emerged as an accurate noninvasive diagnostic tool for ATTR-CM when coupled with appropriate evaluations to rule out light chain amyloidosis [5,6]. However, accurate prediction and pre-test risk stratification remain important challenges, with many patients still being diagnosed in the advanced stages of ATTR-CM [7].

Davies et al. developed a “simple ATTR-CM risk score”, a screening tool that incorporates six commonly measured variables to predict the risk of ATTR-CM in patients with heart failure with preserved and mildly reduced ejection fraction (HFpEF/HFmrEF) [8]. This risk score uses demographic, comorbidity, and echo variables to provide an easily understood and deployable risk stratification tool aimed at calibrating the pre-test risk of disease. Although the score demonstrated satisfactory performance with an area under the receiver operating characteristic curve of 0.84 in validation cohorts, Black participants constituted less than 5% of the derivation and validation cohorts (12 and 13 patients, respectively). A relatively small external validation cohort (*n* = 66) with 37% black patients was used to additionally validate the score, showing promising results. Additionally, female representation in the study was limited to only 83 patients (20%) in the derivation cohort and 44 patients (18%) in the validation cohort. While the majority of patients with ATTR-CM are males, a larger number of female participants is needed when developing such risk prediction tools. For that reason, further validation is necessary for larger and more diverse patient populations, particularly those with a higher representation of black patients and women. Moreover, patients with a left ventricular ejection fraction (LVEF) of less than 40% (HFrEF) were excluded from the derivation and validation of this score. However, recent data suggest that a significant proportion of ATTR-CM patients present with HFrEF, particularly black patients [9].

Incorporation of risk scores and predictive algorithms into clinical workflows and clinical decision support may improve diagnostic yield for broad populations. However, they may reinforce existing biases and not account for differing disease presentations across diverse populations. For that reason, we sought to assess the performance of the ATTR-CM score in predicting 99mTc-PYP scan positivity in our cohort with a more detailed look into its performance across race, sex, and ejection fraction.

## 2. Methods

### 2.1. Ethical Considerations

This study was conducted in accordance with the ethical guidelines outlined by the Cleveland Clinic Institutional Review Board. Patient data were anonymized and treated with strict confidentiality to ensure privacy and compliance with data protection regulations. The signature of informed consent for this data analysis was waived because of the retrospective nature of the study.

### 2.2. Study Design

This study included consecutive patients referred for clinically indicated 99mTc-PYP with single-photon emission computed tomography (SPECT) at the Cleveland Clinic (Cleveland, OH, USA) between January 2012 and January 2020 and who had undergone 2D transthoracic echocardiography within one year of the 99mTc-PYP scan. Patients were included regardless of LVEF, as opposed to the ATTR-CM score original derivation and validation cohorts [8]. A 99mTc-PYP scan was considered positive according to the American Society of Nuclear Cardiology guidelines on cardiac amyloidosis practice points in effect at the time of evaluation. Patients with a positive 99mTc-PYP scan were diagnosed by amyloidosis specialists after the exclusion of light chain amyloidosis (AL) and based on standard guideline-based criteria. Genetic analysis was conducted to assess familial or wild-type status.

The ATTR-CM score was calculated as published by Davies et al. and consisted of the following variables: Age (if 60–69, +2; if 70–79, +3, if ≥80, +4), sex (if male, +2), hypertension diagnosis (if present, −1), LVEF (if <60%, +1), posterior wall thickness (if ≥12 mm, +1), and relative wall thickness (if >0.57, +2). A score of ≥6 was considered high-risk.

### 2.3. Echocardiographic Data

For each patient, data were extracted from the prior transthoracic echocardiogram closest to the 99mTc-PYP scan in the echocardiography lab database. Standard measurements, including left ventricular ejection fraction, posterior wall thickness (PWT), interventricular septal thickness (IVST), and left ventricular end-diastolic diameter (LVEDD), were obtained by certified cardiac sonographers according to the American Society of Echocardiography guidelines and validated by accredited staff cardiologists. Relative wall thickness (RWT) was calculated as (IVST + PWT)/LVEDD, and left ventricular mass (LVM, g) was calculated using the Devereux formula (LVM = 0.8 × [1.04 × ((LVIDd + PWd + IVSd)3 − LVIDd3)] + 0.6) and indexed over body surface area.

### 2.4. Statistical Analysis

Group comparisons were conducted using appropriate statistical tests based on the distributional assumptions of the variables. The normality of continuous variables was assessed using the Shapiro–Wilk and Kolmogorov–Smirnov tests. Two-sample *t*-tests were used for normally distributed continuous variables, while the Wilcoxon rank-sum test was employed for non-normally distributed continuous variables. Categorical variables were compared using either the Pearson χ^2^ test or the Fisher exact test, depending on the expected cell frequencies.

The discrimination performance of the ATTR-CM score was evaluated using the area under the receiver operating characteristic (ROC) curve (AUC). A higher AUC indicates better discrimination ability of the score in predicting 99mTc-PYP scan positivity. Sensitivity, specificity, positive predictive value (PPV), and negative predictive value (NPV) were calculated at various score cutoffs to assess the diagnostic accuracy of the ATTR-CM score. Calibration of the model was assessed using the Hosmer–Lemeshow goodness-of-fit test, where a *p*-value greater than 0.05 indicates consistent calibration with the model.

Subgroup analyses were performed to assess the performance of the ATTR-CM score in specific subgroups based on race, sex, or combinations of both. These analyses involved comparing the AUCs, sensitivity, specificity, PPV, and NPV. Additionally, to account for the inclusion of patients with HFrEF, the performance of the ATTR-CM score was evaluated in both the overall cohort and a subgroup analysis comparing patients with LVEF < 40% and those with LVEF ≥ 40%.

Statistical analysis was performed using SPSS software, and a significance level of 0.05 was used to determine statistical significance. Confidence intervals were reported at the 95% level.

### 2.5. Diversity Information

Our study focuses on tackling healthcare disparities by addressing the limitations of a pre-test risk assessment score that was derived from a predominantly white and male cohort. The objective is to reevaluate the performance of this score in black patients, who are more likely to have the hereditary variant of ATTR-CM, as well as females. The authors of this paper originate from various backgrounds and belong to multiple races/ethnicities, including White, Hispanic, Asian, Middle Eastern, and South Asian. Four of the authors identify as women.

## 3. Results

### 3.1. Baseline Characteristics

**Figure 1:**
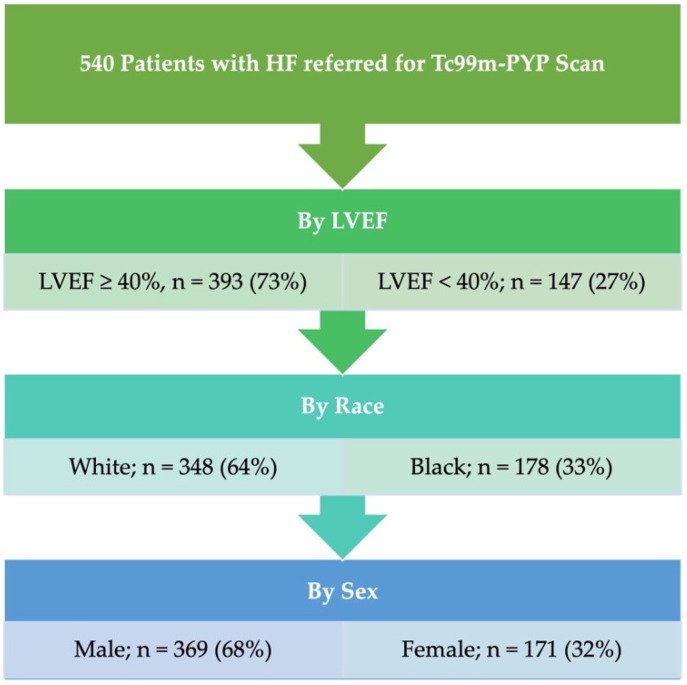
Study flowchart.

The study cohort comprised 540 patients with an overall prevalence of ATTR-CM of 154 (28.5%). The median age was 72 years, and 369 (68.3%) of the subjects were male. Out of the total cohort, 348 patients self-identified (64.4%) as White, while 178 patients (33.0%) self-identified as Black. Regarding LVEF status, 393 patients (72.8%) had an LVEF of 40% or higher (Figure 1). Detailed baseline characteristics are shown in Table 1.

Patients with a positive 99mTc-PYP scan had lower LV end-diastolic diameter (EDD) and LVEF, higher septal and posterior wall thickness (IVST and PWT), as well as higher relative wall thickness (RWT) and left ventricular mass index (LVMi). They also had a significantly higher E/A ratio. Table 2 shows detailed echocardiographic parameters stratified by amyloid status.

**Figure 2:**
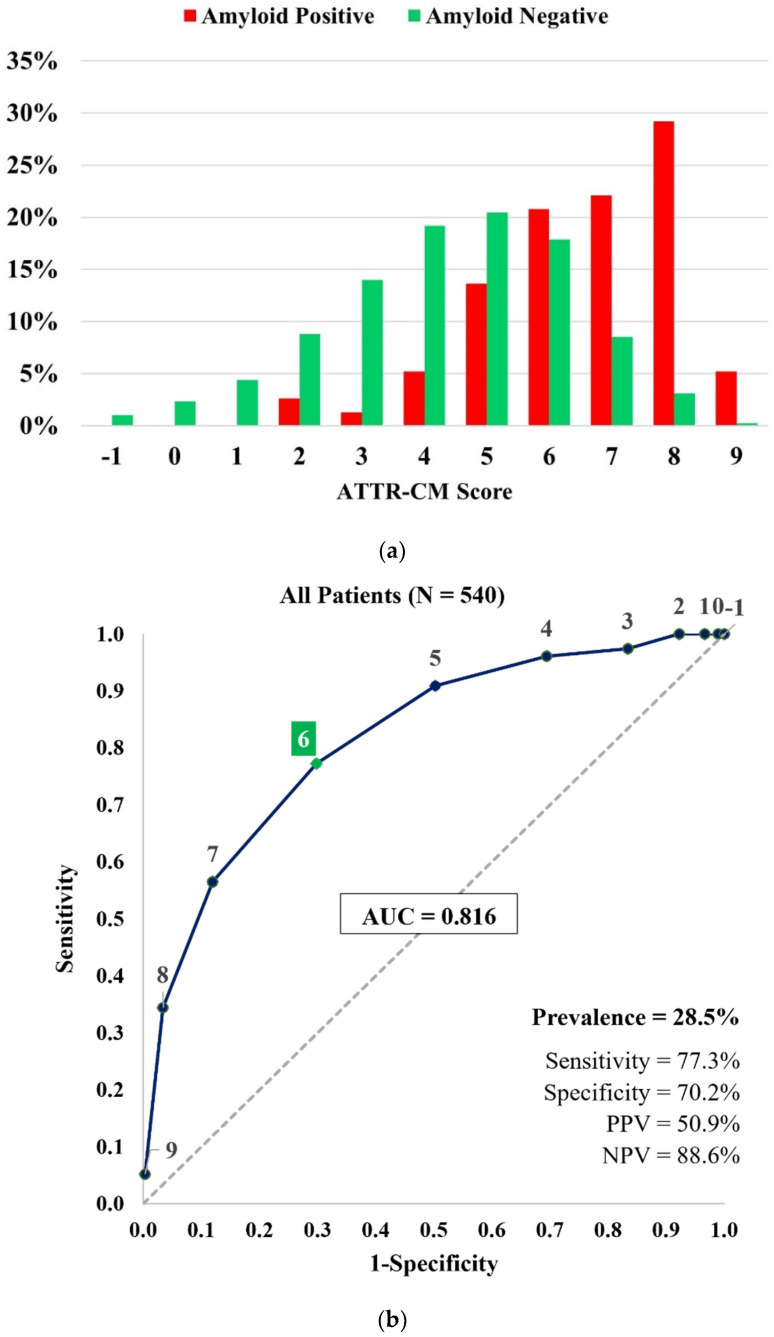
(**a**) Distribution of ATTR-CM scores in the 99mTc-PYP referral cohort (*n* = 540). (**b**) Receiver operating characteristic curve and performance measures of the ATTR-CM score in the 99mTc-PYP referral cohort (*n* = 540). The “high risk” threshold is set at a score of ≥6 to estimate sensitivity, specificity, PPV, and NPV.

When comparing the components of the ATTR-CM score, the difference was significant for each of the variables. Patients with a positive 99mTc-PYP scan were more likely to be older, male, have an LVEF < 60%, posterior wall thickness ≥ 12mm, and relative wall thickness > 0.57. They were also less likely to have a diagnosis of hypertension. Figure 2 shows the distribution of ATTR-CM scores in 99mTc-PYP positive and negative patients and score performance in the entire cohort. The risk score demonstrated an AUC of 0.816, a sensitivity of 77.3%, and an NPV of 88.6% when applied to the entire cohort.

### 3.2. Performance of the ATTR-CM Score by Left Ventricular Ejection Fraction

**Figure 3:**
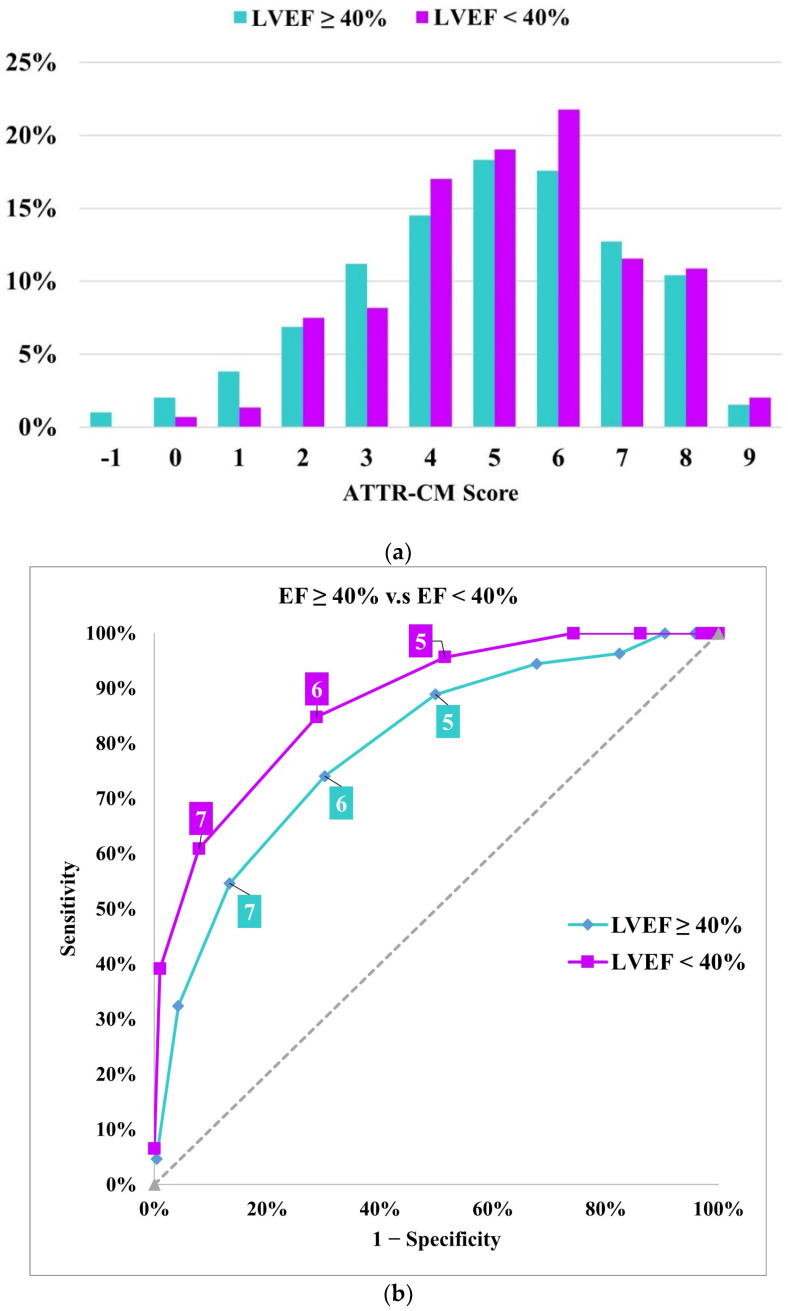
(**a**) Distribution of ATTR-CM scores in the 99mTc-PYP referral cohort stratified by LVEF. (**b**) Receiver operating characteristic curve and performance measures of the Simple ATTR-CM score across LVEF categories. The “high risk” threshold is set at a score of ≥6.

Receiver-operating characteristic curves showed satisfactory performance in both LVEF subgroups, similar to the overall performance of the score, with an AUC of 0.764 in patients with LVEF ≥ 40% and 0.874 in patients with LVEF < 40%. Figure 3 shows the distribution of ATTR-CM scores and the performance of the score by EF subgroups. 

Sensitivity was 74.1% in patients with LVEF ≥ 40% and 84.8% in patients with LVEF < 40%. The negative predictive value was 87.7% in patients with LVEF ≥ 40% and 91.1% in patients with LVEF < 40%. The positive predictive value was 48.2% in patients with LVEF ≥ 40% and 57.4% in patients with LVEF < 40%.

### 3.3. Performance of the ATTR-CM Score by Race and Sex

Black ATTR-CM patients were younger, more often female, and had a higher prevalence of hypertension as compared to White ATTR-CM patients (Supplemental Table S1). Additionally, Black ATTR-CM patients had significantly higher septal, posterior, and relative wall thickness (Supplemental Table S2). ATTR-CM was more often familial in Black patients, as confirmed by genetic studies (49% in Black vs. 8.7% in White) (Table 3).

**Figure 4:**
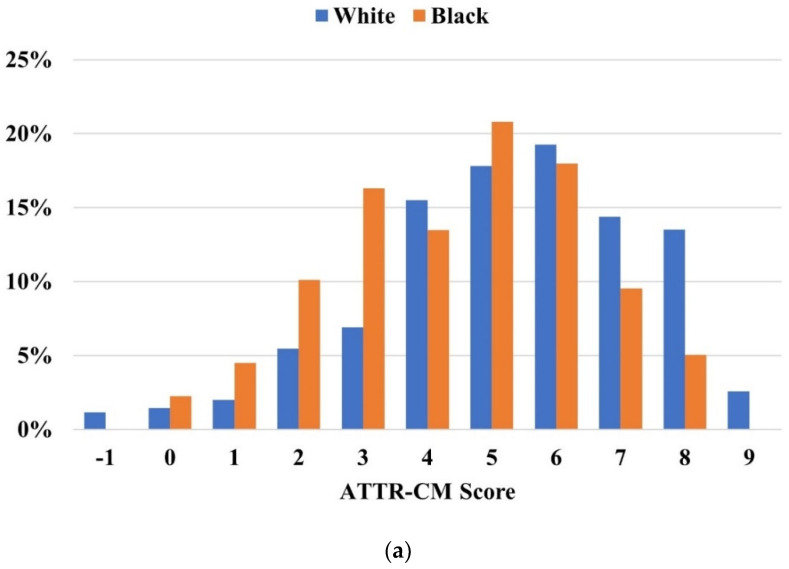

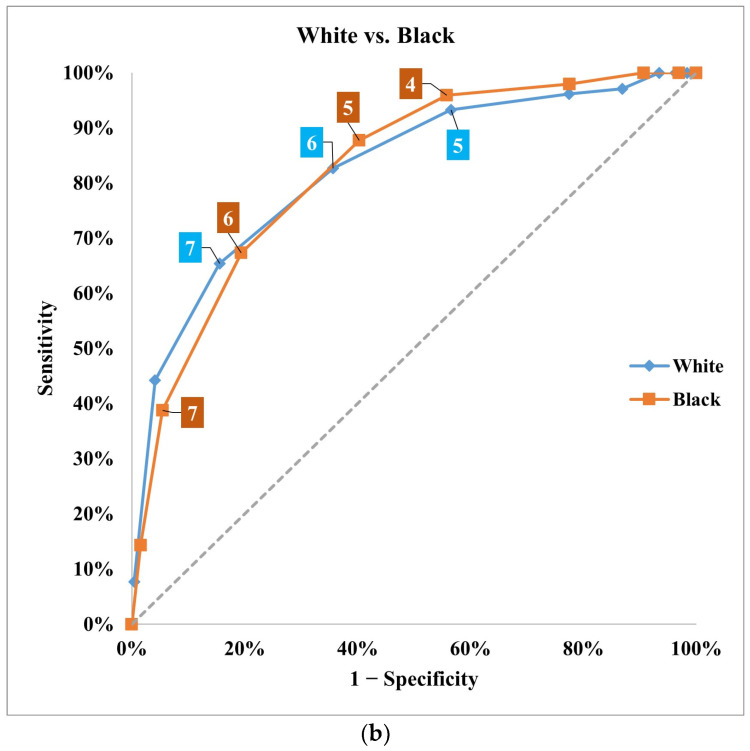
(**a**) Distribution of ATTR-CM scores in the 99mTc-PYP referral cohort stratified by race. (**b**) Receiver operating characteristic curve and performance measures of the Simple ATTR-CM score across races. The “high risk” threshold is set at a score of ≥6.

**Figure 5:**
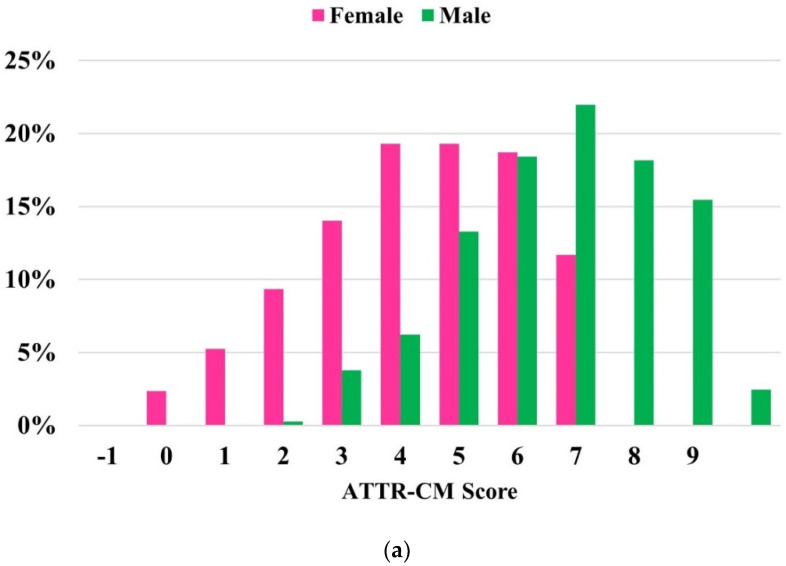

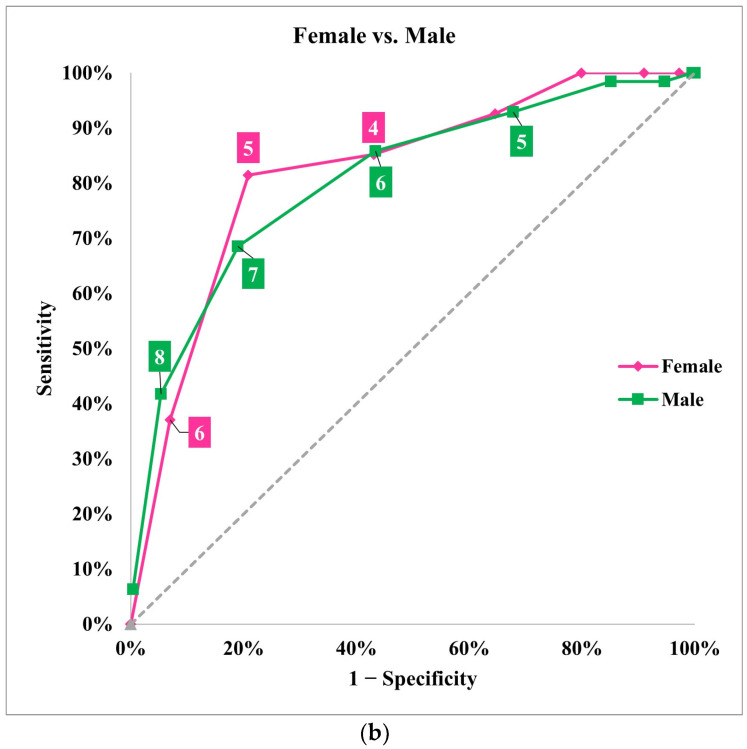
(**a**) Distribution of ATTR-CM scores in the 99mTc-PYP referral cohort stratified by sex. (**b**) Receiver operating characteristic curve and performance measures of the Simple ATTR-CM score across sex categories. The “high risk” threshold is set at a score of ≥6.

The discriminatory power of the ATTR-CM score remained consistent across both Black and White patients, as indicated by an AUC of 0.824 in both groups (Figure 4). Similarly, the score exhibited robust discriminative ability in both males and females, with AUC values of 0.806 and 0.820, respectively (Figure 5).

When utilizing a cutoff score of 6 for high-risk classification, the sensitivity of the ATTR-CM score was relatively lower in Black patients (67.3%) (Table 4). More importantly, sensitivity using this cutoff was remarkably low in females (37%) and particularly low in white females (20%) compared to black females (47%). Accordingly, specificity was higher in females (93.1%), with comparable PPV (50%) and NPV (88.7%) (Table 4). This is also observed in Figure 5b, where sensitivity drops remarkably in females when using a cutoff score of 6 as compared to 5. Detailed score performance in females was evaluated using a modified cutoff of 5, which demonstrated a better sensitivity of 81.5% in all females and 70% in White females (Table 4). This modification in the cutoff for females did not cause a significant change in specificity, PPV, or NPV, which remained comparable to score performance in the entire cohort using the original cutoff of 6.

## 4. Discussion

The aim of our study was to understand the performance of a previously validated ATTR-CM risk score [8] in a more diverse cohort of patients and examine differences across race, sex, and LVEF. The ATTR-CM risk score, composed of six simple and easily accessible variables, had demonstrated robust discrimination and calibration, along with favorable sensitivity and negative predictive value (NPV) in the original derivation and validation cohorts. Our results show that (1) the score performs well in HFpEF/HFmrEF but also has good predictive value in patients with HFrEF; (2) despite notable comorbidity, genetic, and presentation differences between White and Black patients as well as lower sensitivity for detecting disease in Black patients, the simple risk score performed well across racial groups; and (3) the score had markedly lower sensitivity in females, which improved with lowering the high-risk score threshold from 6 to 5 in females.

In Davies et al., the referral derivation cohort consisted of 416 patients with an ATTR-CM prevalence of 45% and a median age of 76 [7]. The majority of patients were male (80%) or white (94%). The community validation cohort had 286 patients with better female representation (48% female) but was predominantly White (96%). The external referral validation cohort consisted of 66 patients, among which 23 (37%) were Black. No patients had an LVEF < 40% in accordance with the inclusion criteria of their study. In this study, we present a large, racially diverse referral cohort of 540 patients, with 178 Black patients making up 33% of the total cohort. Additionally, 171 (32%) were females, and 147 (27%) had an LVEF < 40%. 

The first objective was to evaluate the performance of the ATTR-CM score in all patients with suspected heart failure, regardless of their LVEF. Given the prevalence of HFrEF in ATTR-CM (up to one-fourth at initial presentation), a high index of suspicion and accurate risk stratification tools are needed in these patients. Additionally, black patients present more often with HFrEF ATTR-CM, and the exclusion of HFrEF from risk stratification tools might exacerbate disparities in diagnosis and treatment [8]. In this study, we observed high discrimination and accuracy of the ATTR-CM risk score in HFrEF patients, with slightly higher AUC and sensitivity when compared to HFpEF/HFmrEF patients. These findings suggest that the risk score can be appropriately used to screen HF patients for ATTR-CM across the LVEF range.

Our second objective was to assess the performance of the score in a cohort enriched by a higher proportion of Black patients and females. Although the risk score had comparable discrimination, sensitivity was lower when using a cutoff of 6 in Black patients (67%) as compared to White (82%). This is an important finding, given its potential for amplifying racial disparities in ATTR-CM screening. This might be explained by the high prevalence of hypertension in the Black population more broadly, which is also seen in the Black subset of the ATTR-CM cohort, which effectively translates to the reduced importance of hypertension as a discriminatory feature for ATTR-CM in Black patients. Since the risk score deducts one point for comorbid hypertension, lowering the high-risk cutoff to 5 appropriately elevates the sensitivity and NPV of the score among Black patients. 

With respect to sex differences, the risk score showed good discrimination in sex, although sensitivity was markedly lower in females (37%) than in males (sensitivity 89%). Lowering the high-risk cutoff to 5 drastically improved the sensitivity and slightly increased the NPV in females without significantly compromising the specificity and PPV. Interestingly, this might mean that the addition of 2 points for male sex in the risk score is translating to reduced diagnostic accuracy among women. Although the lower sample size and prevalence of ATTR-CM in females may be responsible for lower sensitivity and specificity, this discrepancy does point to a limitation of the score, which suggests the potential benefit of a sex-specific cutoff to address this issue. Overall, these findings suggest that a modified high-risk cutoff of 5 might be more appropriate when using the ATTR-CM risk score in females and in Black patients while retaining the original cutoff of 6 in males and non-Black patients, depending on what would be considered optimal sensitivity and specificity for ATTR-CM screening. 

Given the ongoing concern of late presentation and missed diagnosis in patients with ATTR-CM, the simple and easily deployable risk tool developed by Davies and colleagues is to be applauded [4,8]. However, the predictive value of a risk prediction tool is dependent on the cohort used for derivation. Therefore, assessing the performance of such a risk score in diverse populations can help reduce diagnostic biases and enhance its wider applicability. With the welcome acceleration in the development of diagnostic risk modeling, including those employing artificial intelligence and machine learning, special attention to the diversity of the derivation and validation cohorts used to train models is needed [10,11,12]. Further efforts to enhance the integration of risk prediction models into EHR-based alerting, though welcome, should consider race and sex-specific thresholds. 

## 5. Limitations

Given that this study represents the retrospective evaluation of a single tertiary amyloidosis center, there are inherent limitations. Additionally, patterns of referral for ATTR-CM screening continue to evolve over time with increasing awareness of the disease. Risk prediction models should continue to be revisited as temporal changes in referral patterns occur.

## 6. Conclusions

While there is a need to scale and build risk scoring into clinical decision-support in order to prevent diagnostic delay, our study suggests that accounting for sex and race-specific differences in risk modeling could lead to higher yield screening and reduce disparities in the diagnosis and treatment of lesser-known cardiomyopathies. Furthermore, a previously validated risk score has good predictive value across the LVEF spectrum, which may expand the population of ATTR-CM patients to whom it may be applied. 

## 7. Clinical Perspectives

In this retrospective analysis of a large cardiac amyloidosis registry, this study utilized a more diverse cohort of patients screened for ATTR-CM to evaluate differences across sex and race in the performance of a risk prediction tool that was derived from a predominantly male and Caucasian cohort of patients screened for ATTR-CM. There may be a need for sex and race-specific cut points when utilizing predictive models in order to avoid the systematic biases built into disease detection.

With increasing interest in utilizing learning algorithms and risk modeling to inform disease prediction and clinical decision support, scrutiny is needed on the cohorts used for derivation and the target population intended for screening. Sex and race differences in disease presentation may influence the performance of risk prediction tools.

## Figures and Tables

**Table 1:** Baseline demographic and clinical characteristics of the 99mTc-PYP scan referral cohort. Data are presented as frequency (%) or median ± standard deviation (* *p* < 0.05).

		All (N = 540)	TcPYP-Negative (N = 386)	TcPYP-Positive (N = 154)	*p*
Age *		72 ± 12.0	71 ± 12.2	80 ± 9.5	<0.001 *
Sex *	Male	369 (68.3%)	242 (62.7%)	127 (82.5%)	<0.001 *
	Female	171 (31.7%)	144 (37.3%)	27 (17.5%)	
Race	White	348 (64.4%)	244 (63.2%)	104 (67.5%)	0.17
	Black	178 (33.0%)	129 (33.4%)	49 (31.8%)	
	Other	14 (2.6%)	13 (3.4%)	1 (0.6%)	
LVEF	<40%	147 (27.2%)	101 (26.2%)	46 (29.9%)	0.38
	≥40%	393 (72.8%)	285 (73.8%)	108 (70.1%)	
BMI *	<25 kg/m^2^	135 (25.0%)	93 (24.1%)	42 (27.3%)	0.045 *
	25–30 kg/m^2^	198 (36.7%)	139 (36.0%)	68 (44.2%)	
	>30 kg/m^2^	207 (38.3%)	154 (39.9%)	44 (28.6%)	
Smoking (current or former)		299 (55.4%)	210 (54.4%)	81 (52.6%)	0.52
Hypertension *		453 (83.9%)	333 (86.3%)	120 (77.9%)	0.017 *
Diabetes *		216 (40.0%)	179 (46.4%)	37 (24.0%)	<0.001 *
Dyslipidemia		382 (70.7%)	265 (68.7%)	117 (76.0%)	0.91
Atrial Fibrillation/Flutter		258 (47.8%)	174 (45.1%)	84 (54.5%)	0.14
History of MI		75 (13.9%)	56 (14.5%)	19 (12.3%)	0.51
History of Stroke		70 (13.0%)	50 (13.0%)	20 (13.0%)	0.99
Coronary Artery Disease		267 (49.4%)	190 (49.2%)	77 (50.0%)	0.94
Any Device		115 (21.3%)	74 (19.2%)	41 (26.6%)	0.39
Gilmore Score	1	192 (35.6%)	140 (39.1%)	52 (35.4%)	0.54
	2	175 (32.4%)	125 (34.9%)	50 (34.0%)	
	3	138 (25.6%)	93 (26.0%)	45 (30.6%)	
1-year mortality		75 (13.9%)	47 (12.2%)	28 (18.2%)	0.07
Creatinine at Baseline (mg/dL)		1.36 ± 1.23	1.35 ± 1.34	1.4 ± 0.85	0.021
Hemoglobin at Baseline (g/dL)		12.3 ± 523.53	12.2 ± 2.3	12.6 ± 2.0	<0.001
NT-ProBNP (pg/mL)		2536.5 ± 9042	2015 ± 9811	3761 ± 6957	0.938
Troponin T (ng/mL)		0.49 ± 2.67	0.48 ± 2.7	0.5 ± 2.3	0.445

**Table 2:** Baseline echocardiographic variables in the 99mTc-PYP scan referral cohort. Data are presented as median ± standard deviation (*p*). SD; standard deviation; LV; left ventricle, EDD; end-diastolic diameter, ESD; end-systolic diameter, IVST; interventricular septal thickness, PWT; posterior wall thickness, RWT; relative wall thickness, EDV; end-diastolic volume, ESV; end-systolic volume, LA; left atrium, RA; right atrium, RVSP; right ventricular systolic pressure.

	All (N = 540)		TcPYP-Negative (N = 386)		TcPYP-Positive (N = 154)		
	*N*	Median ± SD	*N*	Median ± SD	*N*	Median ± SD	*p*
LV ESD (cm)	532	3.4 ± 0.96	382	3.4 ± 1.0	150	3.4 ± 0.8	0.185
LV EDD (cm)	540	4.7 ± 0.84	386	4.8 ± 0.9	154	4.4 ± 0.7	<0.001
IVST (cm)	540	1.4 ± 0.4	386	1.3 ± 0.4	154	1.6 ± 0.3	<0.001
PWT (cm)	540	1.3 ± 0.34	386	1.2 ± 0.3	154	1.5 ± 0.3	<0.001
RWT	540	0.58 ± 0.22	386	0.53 ± 0.20	154	0.69 ± 0.22	<0.001
LV EDV (mL)	408	102.5 ± 47.8	290	103.6 ± 51.2	118	98.9 ± 37.0	0.010
LV ESV (mL)	408	45.7 ± 37.8	290	44.6 ± 41.5	118	48.1 ± 26.5	0.083
Stroke Volume (mL)	540	43.2 ± 29.0	386	43.9 ± 29.8	154	41.7 ± 26.8	0.007
LVEF (%)	540	52.4 ± 14.6	386	53.9 ± 14.7	154	48.0 ± 14.2	0.007
LV Mass Index (g/m^2^)	186	136.8 ± 47.6	132	126.9 ± 44.0	54	150.4 ± 50.8	<0.001
LA Volume (mL)	512	91.9 ± 38.8	365	91.9 ± 41.3	147	92.7 ± 31.6	0.811
LA Volume Index (mL/m^2^)	539	44.3 ± 21.8	386	44.1 ± 23.4	153	45.2 ± 17.1	0.793
LA Area (cm^2^)	268	25.1 ± 7.0	194	25.0 ± 7.3	74	25.9 ± 6.1	0.384
RA Area (cm^2^)	277	21.2 ± 7.6	197	20.3 ± 7.7	80	23.7 ± 7.3	0.017
E/A ratio	337	1.57 ± 1.14	251	1.39 ± 1.1	86	2.29 ± 1.1	<0.001
e’ Septal (cm/s)	399	0.050 ± 0.018	282	0.056 ± 0.019	117	0.05 ± 0.015	<0.001
e’ Lateral (cm/s)	404	0.070 ± 0.054	287	0.070 ± 0.026	117	0.06 ± 0.091	0.295
E/e’ Septal	399	15.4 ± 10.6	282	14.9 ± 10.1	117	17.2 ± 11.7	0.638
E/e’ Lateral	404	11.8 ± 8.8	287	11.4 ± 8.1	117	12.4 ± 10.2	0.760
E/e’ Average	414	13.6 ± 9.3	294	13.3 ± 8.8	120	14.9 ± 10.6	0.798
RVSP (mmHg)	444	41.6 ± 15.2	312	42.7 ± 16.3	132	40.2 ± 11.8	0.010
s’ (cm/s)	382	10.0 ± 3.3	279	10.8 ± 3.1	103	9.0 ± 3.3	<0.001

**Table 3:** Subtypes of TTR Amyloidosis in 99mTc-PYP-positive patients.

Amyloid Type	All	Black	White	*p*
Wild Type	76 (49.4%)	12 (24.5%)	64 (61.5%)	<0.001
Familial	34 (22.1%)	24 (49.0%)	9 (8.7%)	
Unknown (no genetic study)	44 (28.6%)	13 (26.5%)	31 (29.8%)	
Total	154	49	104	

**Table 4:** Performance measures of the ATTR-CM Score in sex and race subgroups. Differences in performance measures of the ATTR-CM Score in sex and race subgroups when using the suggested high-risk cutoff of 6 and modified cutoff of 5. Bold: highlight sensitivity, Underline: highlight sensitivity in black patients and in women.

	White	Black	Male	Female	All
N	348	178	369	171	540
Prevalence of ATTR-CM	29.9%	27.5%	34.4%	15.8%	28.5%
For a Score cutoff ≥ 6					
Sensitivity	**82.7%**	**67.3%**	**85.8%**	**37.0%**	**77.3%**
Specificity	64.3%	80.6%	56.6%	93.1%	70.2%
PPV	49.7%	56.9%	50.9%	50.0%	50.9%
NPV	89.7%	86.7%	88.4%	88.7%	88.6%
For a Score cutoff ≥ 5					
Sensitivity	**93.3%**	**87.8%**	**92.9%**	**81.5%**	**90.9%**
Specificity	43.4%	59.7%	32.2%	79.2%	49.7%
PPV	41.3%	45.3%	41.8%	42.3%	41.9%
NPV	93.8%	92.8%	89.7%	95.8%	93.2%

## Data Availability

The data utilized in this study are from the Cleveland Clinic and are not available for public disclosure in accordance with institutional policy.

## Supplementary Materials

The following supporting information can be downloaded at: https://www.mdpi.com/article/10.3390/jcm13206150/s1, Table S1: Baseline demographic and clinical characteristics in 99mTc-PYP-positive racial subgroups; Table S2: Differences in the ATTR-CM Score component variables between Amyloid-Positive and Amyloid-Negative patients; Table S3: Differences in the ATTR-CM Score component variables between wild-type ATTR-CM and Familial ATTR-CM patients; Table S4: Differences in the ATTR-CM Score component variables between female and male patients; Figure S1: Distribution of ATTR-CM scores in Amyloid-Positive patients stratified by ATTR-CM Subtype (in patients who underwent genetic study).

## Abbreviations

ATTR-CM	Transthyretin amyloid cardiomyopathy
HF	Heart failure
99mTc-PYP	Technetium 99m pyrophosphate
HFpEF	Heart failure with preserved ejection fraction
HFmrEF	Heart failure with mildly reduced ejection fraction
HFrEF	Heart failure with reduced ejection fraction
LVEF	Left ventricular ejection fraction
SPECT	Single-photon emission computed tomography
AL	Light chain amyloidosis
ROC	Receiver operating characteristic
AUC	Area under the curve
PPV	Positive predictive value
NPV	Negative predictive value
EDD	end-diastolic diameter
IVST	Septal wall thickness
PWT	Posterior wall thickness
RWT	Relative wall thickness
LVMi	Left ventricular mass index
AUC	Area under the curve
NPV	Negative predictive value
PPV	Positive predictive value
EHR	Electronic Health Record
