# Supplementary material for: Race, Sex, and Ejection Fraction-Based Differences in Transthyretin Amyloid Cardiomyopathy (ATTR-CM) Risk Prediction
Source: J Clin Med. 2024 Oct 16;13(20):6150. doi: 10.3390/jcm13206150 (PMC11508786; doi:10.3390/jcm13206150)
Supplement: Supplementary file 1 [file jcm-13-06150-s001.zip › jcm-3066422-supplementary.pdf]

		White<br>N = 104 (68%)	Black<br>N = 49 (32%)	p
**Age**		**81.0 ± 9.0%**	**76.0 ± 8.9%**	**0.022**
**Sex**	**Male**	**94 (90.4%)**	**32 (65.3%)**	**0.001**
	**Female**	**10 (9.6%)**	**17 (34.7%)**	
**EF**	**< 40%**	31 (29.8%)	14 (28.6%)	
	**≥ 40%**	73 (70.2%)	35 (71.4%)	0.303
**BMI**	**<25**	27 (26.0%)	15 (30.6%)	0.604
	**25-30**	121 (34.8%)	70 (39.3%)	
	**>30**	146 (42.0%)	56 (31.5%)	
**Smoking (current or former)**		59 (56.7%)	22 (44.9%)	0.537
**Hypertension**		**73 (70.2%)**	**46 (93.9%)**	**0.004**
**Diabetes**		22 (21.2%)	15 (30.6%)	0.377
**Dyslipidemia**		78 (75.0%)	38 (77.6%)	0.804
**Atrial Fibrillation/Flutter**		62 (59.6%)	22 (44.9%)	0.554
**History of MI**		**14 (13.5%)**	**4 (8.2%)**	**0.018**
**History of Stroke**		**8 (7.7%)**	**12 (24.5%)**	**0.015**
**History of CAD**		54 (60.0%)	22 (56.4%)	0.657
**History of PCI**		20 (22.5%)	6 (16.2%)	0.642
**Any Device**		28 (38.9%)	13 (44.8%)	0.582
**Gilmore Score**[9]	**1**	36 (35.6%)	15 (33.3%)	0.750
	**2**	34 (33.7%)	16 (35.6%)	
	**3**	31 (30.7%)	14 (31.1%)	
**CHADS-VASC ≥2**		98 (94.2%)	47 (95.9%)	0.188
**1-year mortality**		20 ± 19.2%	8 ± 16.3%	0.814
**Creatinine at Baseline (mg/dL)**		1.4 ± 0.6	1.5 ± 1.2	0.096
**Hemoglobin at Baseline (g/dL)**		12.6 ± 2.1	12.6 ± 1.7	0.960
**NT-ProBNP (pg/ml)**		3781.5 ± 6778.2	3296.0 ± 7460.6)	0.874
**TnT (ng/ml)**		0.24 ± 2.2	1.4 ± 2.6	0.219

Supp. Table S1. Baseline demographic and clinical characteristics in 99mTc-PYP-positive racial subgroups. Data are presented as frequency (%) or median ± standard deviation (* p <0.05).

**Score Component**	**Value**	**Amyloid -**	**Amyloid +**	**p**
**Age**	**< 60 (+0)**	78 (20.2%)	8 (5.2%)	**< 0.001**
	**60-69 (+2)**	102 (26.4%)	19 (12.3%)	
	**70-79 (+3)**	116 (30.1%)	49 (31.8%)	
	**≥ 80 (+4)**	90 (23.3%)	78 (50.6%)	
**Sex**	**Male (+2)**	242 (62.7%)	127 (82.5%)	**< 0.001**
**Hypertension**	**Present (-1)**	333 (86.3%)	120 (77.9%)	**0.017**
**EF**	**< 60% (+1)**	273 (70.7%)	128 (83.1%)	**0.003**
**PWT**	**≥ 12**	191 (49.5%)	133 (86.4%)	**< 0.001**
**RWT**	**> 0.57**	157 (40.7%)	121 (78.6%)	**< 0.001**

Supp. Table S2. Differences in the ATTR-CM Score component variables between Amyloid-Positive and Amyloid-Negative patients.

Score Component	Value	Wild Type (n = 76)	Familial (n = 34)	p
Age	**< 60 (+0)**	2 (2.6)	5 (14.7)	**0.005**
	**60-69 (+2)**	11 (14.5)	7 (20.6)	
	**70-79 (+3)**	23 (30.3)	15 (44.1)	
	**≥ 80 (+4)**	40 (52.6)	7 (20.6)	
**Sex**	**Male (+2)**	68 (89.5)	24 (70.6)	**0.013**
**Hypertension**	**Present (-1)**	60 (78.9)	26 (76.5)	0.771
**EF**	**< 60% (+1)**	64 (84.2)	27 (79.4)	0.538
**PWT**	**≥ 12**	65 (85.5)	30 (88.2)	0.702
**RWT**	**> 0.57**	56 (73.7)	27 (79.4)	0.519
**Overall Score**	**Median (SD)**	**7 (1.4)**	**6 (1.6)**	**< 0.001**

Supp. Table S3. Differences in the ATTR-CM Score component variables between wild-type ATTR-CM and Familial ATTR-CM patients. *Patients with wild-type ATTR-CM had a median pre-test score of 7 while patients with Familial ATTR-CM had a median score of 6. Patients with wild-type ATTR-CM were more likely to be in older age categories, and were more likely to be male ($p < 0.05$).*

Score Component	Value	Male	Female	p
Age	< 60 (+0)	57 (15.4%)	29 (17.0%)	0.89
	60-69 (+2)	86 (23.3%)	35 (20.5%)	
	70-79 (+3)	112 (30.4%)	53 (31.0%)	
	≥ 80 (+4)	114 (30.9%)	54 (31.6%)	
Sex	Male (+2)	-	-	-
Hypertension	Present (-1)	301 (81.6%)	152 (88.9%)	**0.031**
EF	< 60% (+1)	294 (79.7%)	107 (62.6%)	**< 0.001**
PWT	≥ 12	234 (63.4%)	90 (52.6%)	0.017
RWT	> 0.57	197 (53.4%)	81 (47.4%)	0.193

Supp. Table S4. Differences in the ATTR-CM Score component variables between female and male patients.

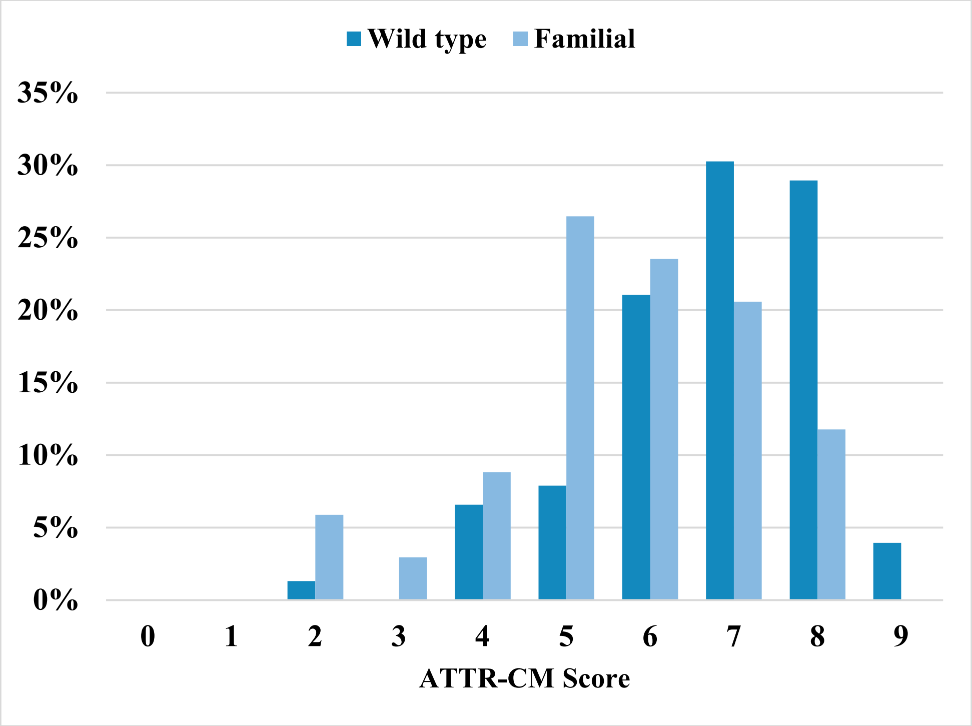

Supp. Figure S1. Distribution of ATTR-CM scores in Amyloid-Positive patients stratified by ATTR-CM Subtype (in patients who underwent genetic study).
